# Medorrhinum

**Published:** 1892-02

**Authors:** Thomas Wildes

**Affiliations:** Kingston, Jamaica


### MEDORRHINUM.

THOMAS WILDES, M. D., KINGSTON, JAMAICA.

On treating of this polycrest, I shall premise by saying that all cases of favus in infants or young children—otherwise *tinea capitis, porrigo, tinea vera,* or scald head—are owing to suppressed gonorrhœa in one or both of the parents, usually, though not invariably, in the father.

I also include in this, *ophthalmia tarsi simplex,* where the margins of the lids are scurfy, scaly, and often angry red, with shedding of the lashes. This red, angry, diseased condition of the skin of the face or scalp may also extend down the back of the neck, down the back, and even under the perineum, involving the genitals.

In one instance, in a girl of eleven years, who had been treated by many physicians and usually with salves and ointments until her general health was much impaired, I found the face mottled with a profusion of red, scurfy sores, the eyelids involved and nearly denuded of lashes, the hairy scalp one diffuse mass of thick, yellow scabs, from beneath which oozed a highly offensive mixture of ichor and sebum. And, passing from the scalp down the back of the neck, down the back, under the perineum, including the genitals, and extending upon the pubes, was a fiery-red band as broad as the child's hand, oozing a pale yellow serum which caused her clothing to adhere to her body.

This was a case where Iris-versicolor, Lycopodium, Graphite, Sulphur, and many other remedies, would ordinarily be used, often with great benefit, but never to a cure. I boldly told the mother that I would cure the case, but that it would surely get much worse for the first three months. This latter she did not object to. I gave one dose of Medorrhinum$^{cm}$ (Swan), on the child's tongue, and a vial of Sac-lac. in pellets, to be taken every night, with orders to return in one month. The external appearance of the case grew rapidly worse, sure enough; but the child's appetite, sleep, and general health steadily improved, and in nine months she was utterly well, and continued so during the two years that I watched the case, in which time she developed as a woman without any abnormal symptoms.

My first cure of the kind was in a child of about six years who was horribly disfigured by tinea capitis, the scalp being a mass of dense scabs exuding fœtid ichor, and the only semblance of hair being a few distorted stumps of roots ending in withered points instead of hair. Her parents actually wished her dead, and openly said so. She had been afflicted since an infant, was steadily under medical treatment, and considered hopeless. One dose cured her in a few months, and, more than that, she is now a healthy, happy young lady of twenty years, with a beautiful and luxuriant head of golden chestnut hair. She is the brightest and most talented in a family of seven very bright children, the youngest of which is now twelve. She graduated from a well-known ladies' seminary, with the highest honors of her class.

Many able writers state that they deem an internal treatment of favus both useless and ineffectual. But before they reiterate the statement I trust they will give one dose of Medorrhinum$^{cm}$, and see each of their cases again at the end of a year.

Others claim that "favus is a disease of the lower classes, attacking only those who pay no attention to cleanliness." This also is a mistake.

Favus, when derived from the father, is liable, if suppressed, to end in hydrocephalus, in capillary bronchitis, in serious teething diarrhœas, in cholera infantum, or some such ailments, often resulting in death. When derived from the grandfather, consumption and other lingering diseases more often follow.

The close relation, both direct and indirect, between gonorrhœa and phthisis I believe to be sufficiently established to no longer occasion much controversy.

Every obstetrician must have encountered numerous cases where infants of a few days or a few weeks old develop a fiery-red rash about their genitals and bottoms, often accompanied with profound constipation and hard, dry stools, showing that the mucous lining of the bowels is also involved in the eruption. In such cases it is not necessary to ask the father if he has ever had gonorrhœa, but simply give the infant one dose of Medorrhinum$^{cm}$, and instruct the mother to keep the parts well powdered until the rash goes away, for she is sure to repeat at each visit that "baby's dear little water scalds it terribly."

My observations have led to the belief that trichiasis and distichiasis are often due to inherited gonorrhœa. I have treated such cases accordingly, and with good results.

The relation between rheumatism and gonorrhœa, both immediate and remote, is too patent to require elucidation here. In every family where rheumatic ailments, and I may also add lung troubles, are prominent, I am always prepared to say that the grandfather or the great-grandfather could a tale unfold that would harrow up the soul. The latent elements are thus in the blood of the offspring, and it only requires the necessary exposure or other inspiring cause, for disease action to commence. In acute cases of rheumatism Medorrhinum is contra-indicated because of the violent aggravation it produces. In chronic cases it is ultimately curative.

Many physicians must have noticed the close relation between rheumatism or gout, paralysis, phthisis, and insanity—the four sometimes occurring in one family or their cousins. Now the problem is, how much of this is due to inherited gonorrhœa in cases where no definite evidence exists of a syphilitic lesion? I claim that either with or without the syphilitic taint in any given case, the basis of origin of these diseases is gonorrhœa. And may it not be that this gonorrhœa taint is what Hahnemann described as psora—that hydra-headed monster which has baffled so many in their efforts to understand its genus and species, and thus caused them, honest men though they often are, to turn against Homœopathy and its tenets?

And now a word concerning the original, simon-pure, daddy of all evils, gonorrhœa in its original state. There are about twenty varieties, from the most simple and bland to the most active and virulent; hence they do not and cannot all affect the human system in the malign and permanent manner as above stated: nor can they all be cured by the same treatment. I have cured many cases rapidly with one dose of Medorrhinum, and the same remedy in other cases has produced such violent aggravations and such grave constitutional disturbances that its use has occasioned me only regrets. Hence I have long ago ceased to use it in any case of primary gonorrhœa.

And I may add, that for fear of results, I have never used it in the acute exanthematic stage of scarlatina, because I attribute the virulence and malignity of some scarlatina cases to latent gonorrhœa in the father, and not to any fault of the doctor. Let the reader picture to his mind the appearance of the tonsils and of the whole palatine arch in a malignant case of scarlatina, and then draw the parallel between that and the appearance of the derma in favus, ophthalmia tarsi, or any angry eczema.

Pneumonia, pleuritis, peritonitis, and such are but too often owing primarily to a gonorrhœal taint, responsive to a proximate cause. Hence one dose of Medorrhinum will often hasten the cure where there is a tendency to relapse. In the acute stage, when the pain is intense, especially at night, and the patient sleepless, Psorinum$^{M}$, a dose every night, acts charmingly.

In dangerous cases of cholera infantum, the patient perhaps sinking and a tendency to marasmus, one dose of Medorrhinum often turns the tide and promotes a cure, the only stumbling block being the difficulty of differentiating between the mild psoric, the gonorrhœal, and the syphilitic, or sycotic taint of the blood of the child. This knowledge comes from experience and observation.

Medorrhinum will be found curative in many cases of obstinate marasmus in children, even after Syphilinum has failed, and usually one dose is sufficient. One case that I cured grew up to be a fine boy, and when past eight years of age he had the audacity to drown himself in the East River at the foot of

Eighty-sixth street, where he had gone to swim with some older boys. He thus spoiled a beautiful case that I was watching with great interest. I began to treat him when he was twenty months old, still nursing from his mother, and when not nursing he was crying night and day. He was very emaciated, and had been given over to die after a consultation by two able men. Then I was called. After a few weeks he rapidly improved to complete recovery. Subsequently, I treated the father for stricture, and obtained a history that confirmed my belief when treating the child, and made me happy, because my experience with Medorrhinum was then slight, and I was gradually learning to place upon the Medorrhinum shelf the diseases which belonged there.

Inherited gonorrhœal taint must be regarded as the leading factor in cases of basilar meningitis of children, and either the inherited or acquired variety is invariably present in cases of idiopathic cerebro-spinal meningitis, whether conjoined or separate. The efficacy of Medorrhinum is doubtful in basilar meningitis. But in cerebro-spinal meningitis one dose of it is surprisingly beneficial after a few doses of Cimicifuga has allayed the first anguish of the case. So soon as convalescence sets in, I then change the Cimicifuga to Lycopodium, with best results.

Able writers have shown that latent gonorrhœa in a man will produce a long train of evils in his wife as serious as that of latent syphilis or sycosis, often eventuating in sterility and even early death. Among these evils may be enumerated ovarian tumors, ovaritis, salpingitis, metritis, para- and endometritis, pelvic peritonitis, etc. Modern gynæcologists recommend operation and enucleation of the affected part. I recommend a few doses of Medorrhinum in cases that manifest no definite syphilitic lesion—when Syphilinum would then take precedence—and wait a few months for the cure which is almost sure to follow. It is worth trying. I have tried it and met with gratifying results in the restoration to health of my patients.

In short, when the gonorrhœal taint is present, give one or more doses of Medorrhinum ; only rarely, if ever, give it in the *acute stages* of a disease.

